# A Review of the Phenotypic Traits Associated with Insect Dispersal Polymorphism, and Experimental Designs for Sorting out Resident and Disperser Phenotypes

**DOI:** 10.3390/insects11040214

**Published:** 2020-03-30

**Authors:** David Renault

**Affiliations:** 1 Université de Rennes 1, CNRS, ECOBIO (Ecosystèmes, Biodiversité, Évolution) UMR 6553, F-35000 Rennes, France; david.renault@univ-rennes1.fr; Tel.: +33-(0)2-2323-6627; 2 Institut Universitaire de France, 1 Rue Descartes, 75231 Paris CEDEX 05, France

**Keywords:** movement, morphology, reproduction, fecundity, wing-dimorphic, wing-monomorphic, mating, range expansion, life-history, hostile matrix

## Abstract

Dispersal represents a key life-history trait with several implications for the fitness of organisms, population dynamics and resilience, local adaptation, meta-population dynamics, range shifting, and biological invasions. Plastic and evolutionary changes of dispersal traits have been intensively studied over the past decades in entomology, in particular in wing-dimorphic insects for which literature reviews are available. Importantly, dispersal polymorphism also exists in wing-monomorphic and wingless insects, and except for butterflies, fewer syntheses are available. In this perspective, by integrating the very latest research in the fast moving field of insect dispersal ecology, this review article provides an overview of our current knowledge of dispersal polymorphism in insects. In a first part, some of the most often used experimental methodologies for the separation of dispersers and residents in wing-monomorphic and wingless insects are presented. Then, the existing knowledge on the morphological and life-history trait differences between resident and disperser phenotypes is synthetized. In a last part, the effects of range expansion on dispersal traits and performance is examined, in particular for insects from range edges and invasion fronts. Finally, some research perspectives are proposed in the last part of the review.

## 1. Introduction

Dispersal is the movement of individuals or populations from the natal (natal or postnatal dispersal, see [1,2] for examples) or breeding (breeding dispersal, see [3] for example) habitat to another breeding habitat [4,5]. This type of animal movement is of particular importance for the stabilization of population demography and dynamics, by balancing the departure of individuals with arrival of other conspecifics (reviewed in [6]). Variations in dispersal success and rates are common among individuals and species, and plastic and evolutionary changes of dispersal traits are often the consequence of spatio-temporal variations in the fitness performance [7]. Variability of dispersal traits can result from different mechanisms, including the risk of inbreeding, which can be deleterious for fitness performance ([8]; inbreeding depression: [9]), kin competition [10] or competition for resources [5]. Dispersal also shapes the spatio-temporal distribution of the genetic diversity of species, in parallel to increasing the proportion of the total genetic diversity of populations [11]. Of note, by reducing genetic drift, dispersal may save populations from local extinction [12], and allow the survival of low density populations (Rescue effect: [13]).

Dispersal capacities are of significant importance for setting and reshuffling the geographic distribution of species. Nowadays, climate change has led to poleward shifts across many taxonomic groups, both on land and in the oceans [14,15], and upwards range shifts within montane ecosystems [16,17]. The extents of these shifts are, at least partially, strongly supported by dispersal capacities of individuals [18,19]. In addition, dispersal enhances the persistence of populations thriving in fluctuating environments [20], and is expected to be more frequent in disturbed or stochastically variable habitats [21,22]. This can be illustrated by the propensity for ballooning dispersal of spider mites, which is increased 5.5 times in specimens from disturbed habitats as compared with their relatives from stable habitats [23]. Importantly, as the increased gene flow allowed by individual dispersal can be random or non-random (see for instance [24]), this can have significant cascading effects on local adaptations of populations. For instance, immigration of insecticide-resistant mosquitoes increases the frequency of resistant alleles within the population, while influxes of susceptible or less adapted mosquitoes would decrease their frequencies (see the review of Miller and Sappington [25]), in turn lowering the resilience of the population. Finally, the ecological importance of insect fluxes also scales up to higher organizational levels, by having for instance significant positive consequences for ecosystem services [26].

All animal species can disperse, but the dispersal rate, the frequency of this event, and the geographical distances that can be covered, can greatly vary among individuals, populations and species. For instance, individuals of a population of the damselfly *Coenagrion mercuriale* can exhibit either short- or long-distance dispersal strategies [27]. Often in animal populations, several individuals remain at their natal site (i.e., philopatric or resident individuals), and a decreasing number of individuals successfully settles outside their natal habitat or home range (i.e., dispersers). Maintaining high dispersal capacity performance can be costly for individuals, and dispersal should be advantageous if benefits (increased fitness of the individual) in the new breeding habitat exceed the costs resulting from dispersal, thus conferring a selective advantage [22] (reviewed in Bonte et al. [28]; but see Hamilton and May [29] who suggested that benefits could be null for dispersing individuals, and only in favor of residents). Consistently, it is often assumed that individuals having the highest dispersal capacities should benefit from the weaker competition in their new breeding habitat [30,31], thus favoring a higher reproduction, growth and developmental performance.

Given the importance of dispersal in driving the ecology and evolution of organisms and populations in the ever-changing environmental conditions, the causes, mechanisms, consequences and costs of dispersal have been studied in a variety of insect models [3,28,32,33,34,35]. Several literature reviews have been published in this field in entomology; yet, these valuable studies are most often species or genus specific [36,37], or focus on the costs of dispersal [28], on trade-offs with reproductive traits [38], or on available methods for the monitoring of insect dispersal [39]. Moreover, since the publication of the book “*Dispersal in Ecology and Evolution*” [6], several new studies have been conducted on this fast moving topic. Thus, the present review, which integrates the more recent advances in the field of insect dispersal ecology, aims at giving an overview of our current knowledge of dispersal polymorphism in wing-dimorphic, monomorphic, wingless, and range-expanding insects. By focusing on active dispersal, i.e., insects dispersing by walking, flying, or swimming, the first part of the article briefly summarizes the different wording and definitions used for defining insects’ dispersal. While working with wing-dimorphic insects eases the differentiation of disperser versus resident individuals, this distinction is less obvious for wing monomorphic insects, and can be even more difficult for non-flying (or poorly flying) ones. The different experimental systems that have been designed for separating disperser and resident insects are thus reviewed, and this second part includes some suggestions of the parameters that could be considered in future studies for manipulating dispersal propensity, rate and success. As insect dispersers are not a random subset of their population, and rather exhibit a suite of traits which offset dispersal costs and increase the probability of dispersal being successful [28,40], the existing knowledge on the morphological and life-history trait differences between resident and disperser phenotypes is synthetized in a third part of the article. Finally, dispersal is increasingly studied in the context of range-expanding insects, including biological invasions, and there is supporting evidence that spatial sorting may contribute to selecting dispersive phenotypes at the front distribution margins. The knowledge of these ecological differences, possibly related to enhanced dispersal capacities at range edges, is discussed in a fourth part.

## 2. The Different Terminologies Used for Describing the Movements of Insects

The geographical scale at which dispersal occurs can be highly variable, and greatly varies among populations and species. The large variation of dispersal distances has often made it difficult to clearly define dispersal from other types of movements [22]. In the literature, different terms are found for describing the main movements of animals, including insects, the most frequent ones being (i) dispersal, (ii) migration, and (iii) movement (see Holyoak et al. [41] for a review). For movement types that do not correspond to dispersal or migration, the following terms are frequently used: Foraging [42,43], homing [44], home range [45], nomadism [46], routine movements [47], or searching behavior [48]. A valuable example of the methodological procedures that can be used for separating the different types of movements can be found in Singh et al. [49].

For insects, migration is often defined according to Kennedy [50], i.e., “migratory behavior is persistent and straightened-out movement effected by the animal’s own locomotory exertions or by its active embarkation on a vehicle. It depends on some temporary inhibition of station-keeping responses, but promotes their eventual disinhibition and recurrence”. Dingle and Drake [51] have completed this definition by summarizing the different migratory patterns that can be observed for different types of organisms (obligate versus facultative migration, timing of the migration, and spatial patterns). Of note, partial migration, i.e., the migration of a portion of the population while the other part of the population remains resident, also exists in insects [52,53]. In a recent review on partial migration of insects, many of the examples used by the authors correspond to dispersal [54], an aspect that the authors recognized themselves (“Other movement ecology researchers might categorize some of the examples we provide in our review as dispersal instead of migration”); this suggests that the definition of migration in entomological studies is not yet clearly fixed.

Dispersal movements can be separated into extra-range dispersal and dispersal. Extra-range dispersal corresponds to “Movement of propagules to regions beyond the boundaries of their [species distribution] range occupied over ecological time” and encompasses six main categories (Leading edge dispersal, corridor, jump dispersal, extreme long-distance dispersal, mass dispersal, and cultivation; [55]). Regarding dispersal, either short or long-distance dispersal [27], several definitions can be found in the literature. Former definitions proposed by Andrewartha and Birch [56] and Southwood [57] posit that dispersal is an individual or a populational diffusion from a source population, which results in an increase of the distance among organisms and lowers aggregation of individuals [58]. Osborne et al. [39] further described dispersal as an “intergenerational spatial movement”. In several studies, the definition and use of the terms dispersal and migration is debated. To try to solve the inconsistent use of these two terms, Dingle [59] suggested the use of “ranging” instead of “dispersal”, and defined “ranging” as “movement over a habitat to explore it, and movement ceases when a suitable home range is located”. However, despite this suggestion, and even if there is no simple definition of dispersal, the most often adopted one in entomological studies presents dispersal as “any movement of individuals or propagules with potential consequences for gene flow across space” [4], thus corresponding to insects moving beyond their neighborhood. In this line, Renault et al. [40] also suggested that dispersal can be functionally discriminated from the two other forms of movement, i.e., routine/home range movements and migration. Specifically, these authors reported that dispersal is being characterized by a distinct frequency over the life of an organism, as compared with the two other movement types, has an effect on gene flow (as defined by Ronce [4]), little link with seasonality, and moderate preparatory physiological changes. It is advised that future entomological studies should use this updated definition of insect dispersal, as in the present review article.

Two main types of dispersal can be observed: (a) Natal or pre-breeding dispersal, when the insect leaves its birth habitat to reach a distinct habitat for reproduction (new breeding site) [1,2], and (b) (post)breeding dispersal, when the individual leaves the habitat where it was reproducing and reaches another reproduction site (movement from one breeding site to another) [3], thus resulting in a relocation of the reproductive habitat of the individual [60]. In both cases, truly active dispersal encompasses three main phases: (i) departure (or emigration), i.e., the decision of the insect to leave a patch, (ii) the transfer phase, corresponding to the path taken by the individual, with the mosaic of biotic and abiotic parameters it will encounter, and (iii) the settlement (or immigration) phase in which the new habitat of the individual is determined [5,40].

## 3. Experimental Methods for Separating Residents from Dispersers in Wing-monomorphic or Wingless Insects under Controlled Conditions

In many populations, most individuals remain at their natal site (resident insects), and few insects successfully move and establish outside their natal (or former breeding) habitat or home range (i.e., dispersers) [4,22]. Dispersal polymorphism has thus been commonly observed in insects, enhancing the performance of (a) fecundity and growth (resident insects) or (b) dispersal capacities (dispersers), in turn resulting into dispersal-related life-history trade-offs (see the example of the butterfly *Melitaea cinxia*, [61], and see [28,62] for reviews).

In wing-dimorphic insects, dispersing and resident phenotypes can be easily separated (dispersers are winged, or they are long-winged, while residents are unwinged, or they are short-winged). Conversely, the distinction of disperser and resident phenotypes, or highly mobile versus less mobile insects, is less evident in wing monomorphic or wingless insect species. As a result, different techniques have been elaborated for their separation, both in the field (landscape scale studies) and under controlled conditions [39]. An overview of the field techniques for measuring insect dispersal can be found in Feldhaar and Schauer [63], with saproxylic species as insect models. Indirect observations can also be conducted with molecular techniques, as for instance done by Suchan et al. [64] who undertook a metabarcoding work on pollen collected from butterflies to determine the source origin of the individuals according to the patches they visited before they were sampled. For experiments under controlled conditions, the methodology based on tethered flight mills, a system that is used for measuring flight behavior and dispersal capacities in insects, has been reviewed recently (see [65,66]). However, there are no comprehensive reviews synthetizing the different experimental systems allowing the assessment of insect dispersal propensity and polymorphism in the laboratory at small spatial scales, in particular for wing-monomorphic insects. In the below sections, the article will thus illustrate for the first time the methodological approaches, other than tethered mills, that can be used for separating resident from disperser phenotypes. After a short presentation of the visual monitoring methods, other experimental systems will be presented into two different parts, depending if they are including or not a hostile matrix to dispersion. In a last part of this section, some suggestions of the experimental conditions that can be manipulated, together with their putative effects on individuals’ phenotypes, are proposed.

### 3.1. Assessing Dispersal Polymorphism Under Controlled Conditions: Photos and Videos

In some insect species, movements of individuals can be used as a reliable proxy of their dispersal capacities, in addition to informing on their walking path characteristics. The movements can be observed by visual monitoring, including photos, or by videos, and the assessment of dispersal tracks can be obtained by regular records of the position of the insect. For instance, video records have been realized for measuring the movements of nymphs and adults of the brown marmorated stink bug *Halyomorpha halys* in Petri dish arenas (diameter: 10 cm) at 25 °C in the dark (fluorescent lights were used for facilitating the records) over a period of 1 h [67]. A similar design has been used by Socha and Zemek [34] who investigated the walking patterns of the bug *Pyrrhocoris apterus*, except that the arena consisted of a white formica cylinder having a diameter of 125 cm and a height of 62 cm. With this experimental design, Socha and Zemek [34] reported that macropterous males and females move on a greater distance than their brachypterous relatives, and exhibited a less tortuous path. Matsumura and Miyatake [68] compared the walking activity of the red flour beetle *Tribolium castaneum* by recording the movements of the insects placed in a Petri dish of 35 mm diameter and 10 mm height over 30 min at 25 °C. A clear cut-off in terms of distance moved was observed among insects, allowing here again to correlate insects’ mobility to walking distances.

While easy to implement for separating resident from disperser phenotypes on the basis of the distance moved and/or dispersal path characteristics, the use of direct observations may have three possible issues. First, the movements of the insects may not always be correlated to their dispersal capacities. Second, by manipulating the insects when they are transferred to the arenas, their subsequent movement behavior may be altered (the same remark applies to the experimental designs presented below). Third, when working with photos and videos, our capacity to (automatically) discriminate individuals from their medium can be a technical issue. Contrasting colored substrate/medium can be used to ease the discrimination, but this procedure is then likely to overstimulate insects’ movements.

### 3.2. Assessing Dispersal Polymorphism under Controlled Conditions: Experimentally Connected Patches in the Absence of Hostile Conditions

In order to mimic dispersal among habitat patches in natural environments, several studies have used experimentally bridged containers (most often, a source and a destination patch; see Table 1 for the description of the main characteristics of some of these experimental studies) [69,70,71,72,73,74,75,76]. This apparatus allows separation of disperser from resident phenotypes over time. This kind of experimental system was formerly designed by Prus [69] for investigating the movement of adult *Tribolium castaneum*, an insect that flies but also disperses by walking [74]. Later, Łomnicki [75] worked with a chain of five containers to examine if the removal of dispersing insects at each generation would result in a decreased dispersal tendency of the remaining individuals, and to subsequently describe the morphological correlates of the dispersing and resident insects. After the seventh generation, and thus the seventh dispersal assay, the proportion of red flour beetles that dispersed from the source container ranged from 0.60 to 0.95 in dispersing insects, and from 0.05 to ca. 0.60 in their resident relatives [75]. By using a chain of five connected containers, this author also observed half-way dispersal, with insects stopping dispersal at the third container.

Working with the fruit fly *Drosophila melanogaster*, Edelsparre et al. [76] used a container-to-container system and demonstrated that foraging and dispersal polymorphism may have co-evolved, i.e., flies having the highest foraging activity also had the highest dispersal tendency. Similar findings were drawn by Tung et al. [77] who subjected the fruit flies to artificial selection for increased dispersal over 33 generations with a system of two containers; these authors proved the efficiency of this design for experimental selection of flies with higher dispersal ability. Moreover, it allowed demonstrating that dispersal was linear over time, as Tung et al. [77] reported that ca. 25% of the fruit flies dispersed after 3 h, and 50% after 6 h, a conclusion which is also in line with the results obtained by Arnold et al. [72] on *T. castaneum*. Importantly, photoperiod, which is not always reported in dispersal studies (Table 1), may have a significant role on flight initiation, as shown by Drury et al. [74] who tested the effect of light on the propensity of the red flour beetles *T. castaneum* to fly.

In sum, by using connected containers, several studies confirmed the existence of dispersal polymorphism in insects, in particular in wing-monomorphic and wingless species. In addition, this apparatus has allowed to select insects with higher movement ability and foraging activity over multiple generations, suggesting that movement ability may have a genetic background. Yet, these investigations may somewhat lack of ecological realism, and this has motived the introduction of a hostile matrix into the experimental design in other studies.

### 3.3. Assessing Dispersal Polymorphism Under Controlled Conditions: Experimental Systems Incorporating Hostile Conditions

In natural environments, dispersers will likely have to cross physical barriers (climb, obstacles, wind, etc.), and will have to deal with different soil substrates that could make walking more difficult. The introduction of a hostile matrix in the dispersal path connecting the two containers (patches) has thus been considered in some experimental systems (see [78]), as it increases the likelihood that movement among patches is indeed dispersal (and not foraging). In this perspective, the selection of an adequate hostile matrix should be supported by the existing knowledge of the biology and ecology of the tested organisms.

Working with *T. castaneum*, Arnold et al. [72] used three containers and designed five experiments, differing in terms of distance among the containers, and angle made by the flexible tube connecting the containers (Table 1) so that the dispersal difficulty was increased. By using these two types of hostile matrix, these authors found that: (a) A higher angle of the connecting tube, i.e., a more difficult dispersal path, lowered the dispersal success of males and females of *T. castaneum*, and (b) the increased length of the tubes connecting the containers increased the duration necessary for reaching the terminal (destination) container. Morrison et al. [79] modified the Arnold’s et al. [72] system by working with two containers whose distance was increased (25, 75, and 175 cm) in order to investigate the effects of short exposures to pyrethroid (long-lasting insecticide-incorporated netting) on the dispersal capabilities of two insect species. For control insects, the matrix was slightly hostile for the red flour beetle, with fewer insects reaching the destination containers when the distance in between the two containers was the highest (175 cm), as compared with the two other tested distances. Conversely this matrix was not hostile for *Rhyzopertha dominica* (no effects of the tested distances on the number of *Rhyzopertha dominica* that reached the destination container). This experimental design allowed to conclude that dispersal ability of the treated insects from both species was lowered in comparison to their control counterparts.

A complementary study was run for the purpose of this article (Appendix A) in order to get a first overview of the variables of potential interest when designing an experiment with two connected containers for assessing dispersal propensity and performance in entomological studies. This investigation conducted with adults of the lesser mealworm *Alphitobius diaperinus* revealed that (i) warmer temperature increases dispersal propensity, (ii) longer duration of the dispersal assay increased the number of beetles that reached the destination container, and (iii) the type of substrate of the source container did not affect dispersal rates. Regarding the hostile matrix, the angle of the tube connecting the two patches reduced the number of beetles that reached the destination container.

In addition to the creation of a hostile matrix connecting the destination and source patches, some studies may require the introduction of biotic cues that will also increase ecological realism. In their study, Fronhofer et al. [78] added a visual, a chemical and an auditory cue as a proxy of predation risk. For the butterfly *P. brassicae*, the cages were connected by a S-shaped corridor having hostile conditions for the insect (Table 1). The same cages and hostile matrix were used for the damselfly *Platycnemis pennipes*. The dispersal of the marsh cricket *Pteronemobius heydenii* was also assessed in this study. In the three experiments, the authors additionally tested the effect of the availability of trophic resources on dispersal propensity, by having a low and a high resource treatments [78]. Altogether, these experimental designs and investigations nicely demonstrated that the availability of trophic resources and the presence of predatory cues are important causes of dispersal initiation in a range of animal models.

Finally, it is also worth mentioning that other experimental systems can be used for separating resident from disperser individuals. For example, the assessment of the flight endurance of the insects subjected to stressful (hostile) conditions has been measured with a vortex system in butterflies, [80,81]. In this procedure, butterflies were assessed individually for mobility performance in a 250 × 100 × 100 mm plastic container; they were acclimated for 30 s before being vortexed for 60 s at 25 °C. The time spent flying for each individual during this stressful (hostile) minute was recorded, and represented a good correlate of dispersal ability, i.e., dispersers were characterized with good abilities to maintain flight in these hostile conditions, while residents had a lower flying performance [82].

### 3.4. Standardization of the Experimental Systems with an Emphasis of Some Important Parameters Affecting Dispersal Results

By browsing the available entomological literature, it appears that we are missing a consensus regarding the type of apparatus that is being used when running investigations on dispersal. As examples, the investigations conducted on the beetle *T. castaneum* and on the fly *D. melanogaster* make evident the different methodological procedures implemented among existing studies (Table 1). The use of a source container (patch), a dispersal path (corridor), and a destination container (patch) are all components of the experimental dispersal apparatus under controlled conditions. However, the number of destination containers, the length of the dispersal path and its degree of hostility for the tested insect, the number of insects in the source container, the duration of the assay, and the presence/absence of food/medium in the containers can greatly vary among studies (Table 1), in spite of the critical effect these factors can have on dispersal propensity and success.

The need for a standardization of the methodologies is emphasized, in particular among investigations realized with the same species or genus, as it would facilitate comparisons among studies and increase the chances of getting more generalizable conclusions on dispersal strategies and their associated drivers. Also, the studies should provide additional methodological information regarding the experimental system and conditions that have been used. Some of the parameters that should be mentioned and that can be manipulated, together with their possible effects on dispersal, are presented in Table 2. Particular attention should be given to the distance among the source and the destination container, as the dispersal path distance and the duration of the assay contribute to determine if the insects will be sorted out according to their mobility (foraging movement, vagrancy) or according to their dispersal capacities (movements that drive gene flow, as defined by Ronce [4]). Moreover, the harshness (permeability) of the dispersal path (or matrix) is an important factor that can modulate both dispersal success and dispersal syndrome characteristics of disperser versus resident organisms, as recently reported in ciliates [83].

The hostile matrix can be of different nature: absence of (trophic) resources, low to null humidity conditions, shaded/exposed/dark dispersal paths, temperature lower/higher than the thermal preferendum of the species, olfactory cues repulsive / attractive for the species, angle and length of the tubes connecting the containers, internal diameter of the connecting tube. A primary “control quality” of the experimental systems designed for sorting out dispersers and residents can be obtained by observing dispersal propensity (the proportion of individuals that leave their natal patch). The dispersal propensity should range from 10% to 50% of the individuals leaving the initial container/patch; in butterflies Stevens et al. [84] reported that dispersal propensity had an average of 34%.

## 4. Main Morphological, Behavioral, Reproductive, and Fecundity Characteristics of Disperser and Resident Insects

Dispersing insects are not a random subset from the population [24,33,60], and these individuals exhibit a suite of morphological, physiological and behavioral traits which offset dispersal costs and increase the probability of successful dispersal [28]. Phenotypic differences between dispersing and resident individuals have been reported in almost all taxonomic groups that have been studied so far, from single-celled organisms to insects to mammals [22,78,104,105,106,107]. Dispersal polymorphism, and more particularly the phenotypic differences between residents and dispersers, largely results from the value of the traits that enable (for instance: Presence of wings, dispersal behavior such as ballooning), enhance (for instance: lower metabolic rate, higher body size, in particular longer legs and wings) and drive (for instance: fitness performance in the considered abiotic environment) dispersal [108]. Enabling traits are not necessarily restricted to the presence/absence of dispersal structures as in the case of wing dimorphic insects, and can result from the development of the appendices associated with an individual’s movements.

Enabling and enhancing traits related to physiological, biochemical, or energy budget aspects, as well as molecular mechanisms of wing polymorphism and the genetic basis of insect dispersal, have been reviewed by several authors [37,109,110,111,112,113]. Even if physiological, molecular and genetic aspects are not considered in this article, it should be kept in mind that behavior, physiology and life-history are covarying traits which have been unified under the concept of pace-of-life-syndrome [114,115].

### 4.1. Effects of Dispersal Polymorphism on the Phenotype of Disperser and Resident Insects

Dispersal differences have been found in between disperser and resident insects from wingless, wing-monomorphic and wing-dimorphic species in correlation with a suite of morphological characteristics. There are several famous examples of dispersal polymorphism related to wing differences in different insect groups, including Coleoptera, Heteroptera, Hemiptera, or Orthoptera, with several species exhibiting a sedentary (resident) and a dispersing morph [109]. In these insects, macropterous (long-winged) individuals generally represent the dispersal morph, as compared with brachypterous (short-winged) individuals. Some examples include *Pyrrhocoris apterus* (Heteroptera: Pyrrhocoridae) [34], *Metrioptera bicolor* (Orthoptera: Tettigoniida) [116] and aphids (Hemiptera) [117]. Importantly, macropterous insects, which thus have the largest wings, will not obligatorily disperse.

Several morphological differences have also been reported for dispersers and residents from wing-monomorphic or wingless insect species. For instance, Steyn et al. [118] found that dispersers of the Mediterranean fruit fly *Ceratitis capitata* are characterized by higher values of the ratios thorax to body mass as compared with their philopatric (resident) relatives. Often, the size of the dispersal structures correlate with dispersal distances that can be covered, as reported for Trichoptera species from the genus *Ecnomus* whose itinerant specimens are characterized by higher wing size and shapes [119]. In the migratory monarch butterfly, flight performance was associated with wing morphology in both males and females: Insects having longer wings and larger wing areas fly over longer distances, while aspect ratio (length/width of the wing) was not discriminating the flying performance of the butterflies [120]. In the red flour beetle *T. castaneum*, while body size was not correlated with movement, leg length of the insect correlates with movement ability, resulting in farther and fastest dispersal of the beetle [35]. In this insect, longer legs are also associated with higher movement motivation and dispersal distance [73].

Landscape structure and habitat loss may affect the proportion of disperser/resident phenotypes in both wing-dimorphic and wing-monomorphic insects [108,121]. Viljur et al. [122] suggested that all of the butterflies they studied from a managed forest could represent dispersal phenotypes. Environmental conditions, either abiotic (temperature, trophic resources) or biotic (predation, parasitism) can also shape the production of winged offspring. In the pea aphids *Acyrthosiphon pisum*, the proportion of winged offspring is increased when the population is exposed to natural enemies; enemies can be predators (ladybird: [123]); or parasitoids (*Aphidius ervi*: [124]). A similar pattern has also been reported when insects are subjected to increased tactile stimulation with conspecifics or other insects [96]. Seasonal variations in the proportion of disperser and resident insects can also occur, as in the tropical seed bug *Jadera aerola* [125]. In this species, short winged individuals are found during late dry season, and the seasonal wing polymorphism has been hypothesized to occur as a response to the shift from one season to another and to the unpredictability of nutrient availability. The resulting increased proportion of winged insects likely increases dispersal rate of the individuals in order to escape from less favorable habitat conditions.

Interestingly, color patterns of the wings have recently been reported as a morphological proxy that could be used for sorting out noctuid moth populations with long dispersal distance capacities (high variation in color patterns among individuals) in comparison to their resident conspecifics (little variation in wing coloration among individuals) [126]. As more variable color patterns have also been found to increase population abundance and stability in noctuid moths [127], changes in color pattern variability along an invasion gradient could strengthen the range expansion dynamics of invasive insects. Additional observations of possible correlations among wing color patterns, thermal tolerance, dispersal motivation and success should be further examined, as it could have several implications for improving our predictions of range expansion in contexts of climate change and habitat fragmentation.

### 4.2. Effects of Dispersal Polymorphism on the Behaviour of Disperser and Resident Insects

In several insect species, movements of dispersers have a tendency to be more straight during dispersal events, as reported in the wing-dimorphic bug *P. apterus* [34]. The macropterous morphs of this insect are characterized by a more efficient dispersal strategy thanks to straighter dispersal paths, higher mean dispersal speed, and lower exploration time, resulting in higher distance moved as compared with brachypterous individuals [34]. Importantly, the main characteristics of the dispersal path may depend on the degree of fragmentation of habitats [128,129].

Differences in dispersal capacity can be supported by distinct behaviors of the insects according to the environmental conditions of their habitats. In some spider species, for example, there is little or no detectable phenotypic difference between dispersers and residents, but the conditions encountered in the natal patch during the juvenile stage influence the dispersal strategy of the individuals. Specifically, some wolf spiders will disperse over long distances using silk threads (dispersers) and others will remain relatively sedentary by only moving over short distances while abseiling along the plants [130]. Social behavior, in correlation with the morphology of the insect, can also have links / consequences on dispersal patterns [131]. In the beetle *Librodor japonicus*, there are three main body sizes in males (small, medium and large males); large males disperse earlier from the source patch than the two other morphs [132]. In parallel, these males are more aggressive, i.e., they fight more frequently than males of smaller sizes who are avoiding contacts (fights). The authors concluded that resources’ allocation differ among the three morphs, with more investment into testes, wings, and mandibles for the small, medium and large *L. japonicus* males, respectively [132]. A similar finding has been reported from the armed beetle *Gnatocerus cornutus*, whose males having a smaller weapon size (lower enlargement of the mandibles) dispersed more and have a higher spermatogeny expenditure than their counterparts having enlarged mandibles [133]; in the latter phenotype, the rate of remating with the same female is higher and likely explains this lower investment in testis size and volume of sperm production. Finally, and somewhat counterintuitively, dispersers may be more prone to predation. In Gerridae for instance, it has been reported that winged individuals have a reduced ability to walk on the surface of the water, which could affect their ability to eat and escape from predators [134].

Altogether, these findings reinforce the need to determine if there are stable associations of behavioral and life-history traits in dispersing and resident individuals from different populations or species (pace-of-life syndrome theory; [114]). In particular, the existence of similar behavior among insects dispersing by hopping, walking, or flying would suggest that underpinning physiological regulatory mechanisms have been evolutionary conserved.

### 4.3. Effects of Dispersal Polymorphism on the Reproduction and Fecundity of Disperser and Resident Insects

As dispersal incurs costs (reviewed in Bonte [28]), the development of dispersal capacities often has counter-effects on other fitness traits. These costs and trade-offs have been particularly studied in wing-dimorphic insects, as they may be easier to report by taking place before the initiation of dispersal movement (cost of the “winged” phenotype). They are thus known as “pre-departure” costs. In winged insects, the higher musculature of the dispersing individuals, in addition to the energy necessary for fuelling the cost of the dispersal flight, may come at the expense of reproduction [135,136,137]. Yet, increased investment into reproduction may occur after the dispersal event, as observed in macropterous beetles [62]. In particular, higher reproductive effort can be observed from insect dispersers whose flight muscles are histolized after the onset of oviposition in the new breeding patch [38], as reported in the beet webworm, *Loxostege sticticalis* Lezed. In this insect, resources from histolysis are reused to sustain fecundity [138].

Several studies reported the existence of a trade-off in between reproduction and flight between and within insect species. For instance, the aphid *Tuberculatus paiki*, which is a good disperser, has a lower wing loading as compared with *Tuberculatus quercicola* (low level of dispersal in comparison to *T. paiki*); the higher body volume of *T. quercicola* most probably contribute to explaining the higher number embryos measured in this species [139]. Within insect species, the flight-reproduction trade-off has been reported from both wing-polymorphic [62,140,141,142] and wing-monomorphic [81,82,143,144] insects, but this pattern has also been refuted in other studies [145]. In the cricket *Gryllus firmus*, which represents one of the model insect often used for running investigations on dispersal costs, small-winged females produce 60% more eggs than their large-winged counterparts over a period of six weeks [146,147]. This author also reported the existence of a negative correlation between flight efficiency and the number of eggs carried by females, in addition to delayed age at the first reproduction in the dispersing phenotypes of the crickets. In the green lacewing *Chrysoperla sinica*, Khuhro et al. [148] found that females’ flight affected longevity and their subsequent fecundity. Similarly, potential fecundity of females of *Choristoneura conflictana* is reduced after a flight, possibly because of egg resorption [149].

A dispersal-reproduction trade-off also exists in wing-dimorphic insects, with mating latency (age at reproduction of males and females) and duration of copulation being affected by dispersal capacities. In the butterfly *Pieris brassicae*, the measured values for these two reproductive parameters are shorter in individuals having a high mobility (dispersers; [82,142]) as compared with those of low mobility [81]. In males of *T. castaneum* selected for higher mobility, copulation durations were shorter, and these individuals also exhibited a lower stimulation of the females during mating [150], and had lower mating success [151]. In the same vein, the size of eggs was higher in females of *T. castaneum* characterized by lower walking activities and lower dispersal distances, but no difference was found for the number of egg laid (oviposition measured over 50 d) as compared with females having higher walking activities [152]. The higher fecundity and longevity of the resident females as compared with those exhibiting the mobile phenotype can in turn result in a higher net reproductive rate, as found in the codling moth *Cydia pomonella* [136].

The dispersal-reproduction trade-off, centered around the oogenesis-flight syndrome, has been discussed in another review of the special issue “The study of insect movement and foraging strategies” [153], and the recent review of Tigreros and Davidowitz [38] focused on the flight-fecundity trade-off. Briefly, on the 68 studies they analysed, covering 51 different insect species, Tigreros and Davidowitz [38] reported an effect of flight on subsequent females’ fecundity in 39 works, no effect in 16 studies, and a positive effect of flight on fecundity in nine studies. Some of these incongruencies may partly result from uncontrolled quality of the meals taken by the insects, in particular by females. Indeed, there is growing evidence that the nature of the available food resources and landscape structure (patch connectivity) can represent important factors driving the dispersal-reproduction trade-off [151,154]. The quality of the diet can additionally affect the exploratory tendency of disperser and resident phenotypes, and can even have reverse effects in males and females [155].

There are more frequent observations of the dispersal-reproduction trade-off in wing-polymorphic insects, and this may be partly explained by the fact that dispersal (flying activity) occurs before the start of reproduction in these animals. Yet, as in wing-monomorphic insects, this pattern can be more equivocal in some species, as for instance in the navel orangeworm, *Amyelois transitella* (Walker) (Lepidoptera: Pyralidae, Phycitini) for which no trade-off between reproductive output and flight was found [156].

In both wing-dimorphic and wing-monomorphic (and wingless) insects, energetic requirements for flight can have transgenerational fitness costs by affecting the quality of the progeny, whose physical condition partly depends on the resource allocation towards reproduction. In *Lygaeus equestris*, winged individuals produce smaller eggs than wingless ones, which results in transgenerational fitness costs, as the progeny of winged individuals are more sensitive to environmental stress, such as starvation [157]. In a study carried out on the wing-monomorphic fruit fly *Drosophila melanogaster*, Roff et al. [32] reported the energetics cost of flight on the subsequent reproduction of the flies, i.e., egg production of the females is reduced when the duration of the flight is increased.

In wing-dimorphic insects, the presence and / or size of the wings can affect mating success by altering the degree of attractiveness of the individual towards its sexual partner [130]. Dispersal morphs, i.e., large-winged insects, of crickets [158,159,160,161] and aphids [135] have lower mating frequencies and success than their short-wing relatives. In the dimorphic thrips species *Hoplothris pedicularis*, wingless males have longer forelegs (measured as the length of fore-femora) than winged males, and this confers a significant advantage during fights [162], allowing them to have more copulations with females. In *Aquaruis remigis* (Heteroptera: Gerridae), wingless males mate more often than winged males [163], and the reduction of the wings in males of *Cavelerius saccharivorus* (Heteroptera: Blissidae) leads to fitness advantages [164], partly because it reduces the time necessary to reach sexual maturity, thus enhancing mating opportunities for small-winged males. Similarly, in the beetle *Callosobruchus maculatus*, most copulations take place between short-winged males and females [165]. However, there are exceptions to these observations, as for instance in the beetles *Ptinella aptera* and *Ptinella errabunda*, and in the moth *Orgyia thyellina* for which macropterous individuals produce more eggs than their wingless counterparts [166].

Finally, an important aspect that needs to be considered when regarding the dispersal-reproduction trade-off is the nature of the factors that drive it. Indeed, this trade-off can result from (i) pre-existing trade-offs at the individual level (pace-of-life syndrome theory, oogenesis-flight syndrome) or from (ii) the energy consumed during dispersal (cost of dispersal). The origin of the trade-off is likely to have important implications for the individual; in the first situation, a part of the dispersal costs have been already “paid”, whereas in the second situation, dispersal costs will only be paid if the insect effectively disperses.

## 5. Range Expansion and Evolution of Insect Dispersal Traits

The ongoing global changes, and more particularly the degradation of habitats and the warming of many areas worldwide, have opened new ecological niches for many insect species that can move poleward and at higher altitudes in mountains [167,168]. Generally, specimens and species who are highly mobile are more likely to extend their range more consequently than specialist and less mobile ones. For instance, the two butterfly species *Hesperia comma* and *Aricia agrestis*, and the two cricket species *Conocephalus discolor* and *Metrioptera roeselii*, had a significant expansion of their range likely facilitated by the high proportion of macropterous insects in their populations [30]. In parallel, the fast growing populations of non-native insects and their accelerating expanding range in invaded geographic zones suggest that traits enhancing dispersal may be favored at expanding range edges. The existing knowledge of the effects of range expansion on dispersal capacities and the ecological consequences for native and non-native insects are reviewed below.

Dispersal promotion in range expanding species, including in the context of biological invasions [40,169,170], has become a hot topic for ecologists. Larger insect dispersers—with higher sizes of femur, thorax, and abdomen—are more likely to reach distant localities during colonization events [171]; body size of insect populations is thus expected to increase during range expansion [172], in association to enhanced dispersal capacities. Consistently, experimental manipulation of weight loaded by the butterfly *Anartia fatima* suggested that the higher mass allocation to the thorax during range expansion may result from selection for increased dispersal capacities [173]. In the carabid beetle *Merizodus soledadinus* invading the subantarctic Kerguelen Islands, individuals from the populations with longer residence times are also characterized by a smaller body size as compared with insects sampled at the invasion front [172,174], and similar body size patterns have been observed from other insect taxa [175,176].

Movement characteristics, including straighter dispersal trajectories [177,178], is likely to be positively selected during range expansion, as reported in *M. soledadinus* whose adults from range margin populations exhibit higher locomotor activity under controlled conditions [179]. In an experiment aiming at simulating the evolution of dispersal along an invasion gradient, Ochocki and Miller [169] revealed that adult beetle *Callosobruchus maculatus* were characterized by higher dispersal distances after 10 generations. Similarly, in adult *T. castaneum* selected for dispersal capacities over eight generations, Weiss-Lehman et al. [180] found that the intrinsic growth rate of the population was decreased, while dispersal ability was increased, in addition to a slight increase of dispersal speed. This experimental study was conducted to simulate the evolution of dispersal traits at invasion fronts, and revealed that variability of the measured traits was also increased in these individuals. In wing-dimorphic insects, range shifting is also associated with an increase of the dispersal capacities of the macropterous individuals from expansion range margins [181]. Moreover, the higher occurrence of macropters of the cricket *Metrioptera roeselii* in recently colonized areas reveals the crucial role played of this insect morph during range expansion [181].

Founder individuals will further share and transmit their genetic background (assortative mating). As this phenomenon repeats at expanding range edges, dispersal traits should be enhanced, generating phenotypic differentiation between individuals from front and core populations [169], also known as the Olympic village effect (see Chuang and Peterson [182] for a review of the phenotypes that have been observed at invasion fronts in insects and other animal models). The promotion of dispersal traits of individuals at the front of the expansion range has led to the theory of spatial sorting [183]. Of note, in North America, dispersal (measured as the duration of the flight of the individuals and the distance covered in 1 h) of the invasive kudzu bugs *Megacopta cribraria* first increases along the invasion gradient, before decreasing in populations from the invasion frontlines of the species [184]. This observation evidences the importance of the presence of suitable habitats for the range expanding populations, in particular in terms of quality of resources, as this can significantly blur any potential body size pattern along the invasion gradient. This is particularly true for insects having a small diet breadth, as in the case of *M. cribraria* [184].

When expanding their range, the few dispersers colonizing new habitats, whose distance from the core population is being progressively increased, should have direct fitness advantages. Recently established individuals should indeed benefit from decreased intra-specific competition. Moreover, because of this low population density in satellite populations, individuals may remain more active, or should disperse, to find mates. As there are fewer mates, these insects must also be highly fertile, and at the range edge of the distribution, the Allee effect may drive the spreading rate of the populations [185,186]. In the main (core) populations, the competitiveness may be a more important trait to be developed, and the performance of the reproduction of the dispersers at the core should be lower as compared with the residents.

In sum, longer legs/wings, higher muscle mass, larger body reserves, and behavior (straighter dispersal paths) are thus likely to quickly evolve in disperser insects from range expanding species, all of these parameters enhancing dispersal performance [177,187,188]. The current literature also suggests that spatial sorting, population density, gene surfing (genes of insects from range expansion edges are more likely to be found in the population than the genes of the insects from core populations) and patch connectivity represent significant forces shaping the evolution of dispersal capacities in range expanding species. Interactions among Allee effects, density-dependent dispersal propensity, and evolution of dispersal capacities and competitiveness along the invasion gradient render the predictions of invasion dynamics complex, but represent promising research avenues.

## 6. Perspectives

While organisms’ dispersal is being increasingly studied, this review article also points out our lack of knowledge of certain aspects of dispersal processes and mechanisms shaping the disperser and resident phenotypes in insects. Personality-dependent dispersal may have several implications for the successful range expansion of native and non-native insects. However, there are few studies that examined insect personality in terms of exploratory behavior and aggressiveness for instance, or behavioral polymorphism, in correlation with dispersal capacities. Interesting findings could emerge from such investigations, and aliment the idea that dispersal and habitat establishment/colonisation could be a two steps process: individuals having a higher aggressiveness establish populations, then followed by joiners with more social personalities [189]. Some examples of the traits that can be considered and the associated experimental designs can be found in Labaude et al. [190] and Tremmel and Müller [191]. A review of the studies on personality in invertebrates has been written by Kralj-Fiser and Schuett [192], and examples and theory can be found in Spiegel et al. [193] and Dahirel at al. [194].

The reproductive status and the reproductive timing of males and females are associated with distinct endocrine status and management of body energy use. The influence of mate availability on dispersal propensity has already been tested in insects. Conversely, there are less investigations that have been designed to compare the dispersal propensity and performance in mated versus unmated adults, and the effects of reproductive timing on dispersal remains to be explored [160]. Such comparisons may provide us with interesting insights into the possible endocrine and physiological mechanisms triggering inter-individual dispersal variability, in addition to get more information on the cascading effects on the subsequent management of body reserves.

In many animals, including insects, the traits supporting dispersal success of the individual are correlated to a series of other morphological, behavioral, and physiological traits; this set of covarying traits is known as a dispersal syndrome [60]. There are two main categories of dispersal syndromes: The ones resulting from the divergent selection of resident and disperser individuals that were not exposed to the same environmental factors, known as the adaptive dispersal syndromes [6], and the dispersal syndromes that emerged as a result of the association of different traits in relation with dispersal capacities, known as the dispersal syndrome trade-off [6]. Dispersal syndromes generally occur to enhance the chances of coping with environmental constraints during dispersal events, as for instance thermal variability (see Colinet et al. [195] for visualizing the effects of thermal variations on insects’ ecology and physiology). Several authors suggested the existence of different phenotypes among core and range populations [171,172,196,197], in part resulting from the different ecological filters the insects encounter during geographic expansion [40]. Yet, the association of traits which altogether form a dispersal syndrome should be given more attention in the future, in particular for invasive insects along their expansion gradients.

Even if our knowledge of the factors that drive dispersal propensity is improving, we now need to increase our understanding of the effects of the landscape structure [108] on dispersal syndromes, and more particularly improve our understanding of the effects of connectivity of patches, including solitary sites, on these syndromes. In the field, functional connectivity among habitats and the degree of habitat disturbance may shape dispersal distances and performance. For instance, the models proposed by Karisto and Kisdi [198] suggest that connectivity of suitable habitats for the insect determines the nature of the dispersal (local versus global dispersal); future works should now examine how much connectivity could represent an evolutionary force driving the suite of traits of resident and disperser phenotypes.

## Figures and Tables

**Table 1:** Published examples of some experimental systems based on connected patches for studying insects’ dispersal under controlled conditions. The main characteristics of the source and destination containers (patches), the tube (dispersal corridor/pathway) connecting the two containers, and the environmental conditions are presented, in addition to the biological model and observation procedure used for the assessment of dispersal. The presence/absence of a hostile matrix is specified; when present, the main characteristics that made the matrix hostile is summarized. The last column mentions the source article. *Ø: absence of information in the published article.*

Source Container • Diameter; Height; or Volume • Type of Medium	Destination Container • Diameter; Height; or Volume • Type of Medium	Tube • Type • Inner Diameter [ID] • Length	Environmental Conditions • Temperature (°C) • RH (%) Photoperiod	Assessment of Dispersal	Additional Comments	Hostile Matrix	References
• Ø; Ø • 95% wheat flour and 5% yeast	• Ø; Ø • absence of medium	• Plastic • ID 4.5 mm • L Ø	• 29 °C • 75%	Every 24 h over a 10-day period	• Biological model: Red flour beetle, *Tribolium castaneum*	No	[69]
• Ø; Ø • 8000 mg of unsifted flour	• Ø; Ø • 4000 mg of unsifted flour	• Ø • Ø • Ø	• 28 °C • Ø • Constant light	After 15 days	• Biological model: Flour beetle *Tribolium brevicornis*	No	[71]
• 50 mm; 80 mm • 20 g of 95% wheat flour and 5% yeast or absence of medium (depending on the experiment)	• 30 mm; 70 mm • absence of medium	• Glass • ID 4 mm • L Ø	• 29 °C • 70%	After 5 weeks (the time necessary to obtain imagoes that could disperse)	• Biological model: Red flour beetle, *Tribolium castaneum* • Suite of 5 connected containers	No	[75]
• 50 mL • 2 mL of rearing media or agar	• 50 mL • 2 mL of rearing media or agar	• Plastic (1 mL pipette tip) • ID Ø • L 70 mm	• 24 ± 2 °C • 70 ± 5%	After 6 h in 32 5- to 7-day-old flies for each assay	• Biological model: Fruit fly, *Drosophila melanogaster*	No	[76]
• 11 mm; 16 mm • Empty or with 20 mL banana-jaggery medium in the source	• 11 mm; 16 mm • Wet cotton (for moisture)	• Plastic • ID 1 mm • L 2 m (but increased regularly during the experiment up to 10 m)	• 25 °C • Ø	After 6 h	• Biological model: Fruit fly, *Drosophila melanogaster*	No	[77]
• 57 mm; 44 mm • 15 g of flour	• 57 mm; 44 mm • 15 g of flour	• Plastic • ID 4 mm • L from 70 to 620 mm	• 29.5 ± 1 °C • 40%–60% • L12:D12	Twice a day over four days	• Biological model: Red flour beetle, *Tribolium castaneum*. • Suite of 3 connected containers (filter paper only as the medium in the intermediate container)	• Distance among the containers (70, 120, 165, 310, and 620 mm) • Angle of 4, 8, 16, 24 and 55° for the tube connecting the containers	[72]
• 200 m^3^ • Vegetation of low high height • Presence of a water pond (25 L plastic container, 60 × 39 × 16 cm) • Absence of food supply or 2 feeding flowerpots and host plant (fresh cabbages)	• 200 m^3^ • Vegetation of low eight • Presence of a water pond (25 L plastic container, 60 × 39 × 16 cm)	• Ø • Ø • L 19 m	• Ø • Ø • Ø	Dispersal assessed after 4 days, with daily observations	• Biological model: Large white butterfly *Pieris brassicae*	• Narrow S-shaped dispersal corridor, dark and warm • Resource limitation • Predatory cue (visual and olfactory = toads; olfactory cue = 2 crushed butterflies in a tube)	[78]
• 200 m^3^ • Vegetation of low eight • Presence of a water pond (25 L plastic container, 60 × 39 × 16 cm) • Low (cage with natural insect community) or high (adding of approx. 100 fruit flies and a fruit mixture with approx. 200 pupae) resources treatment. treatment.	• 200 m^3^ • Vegetation of high eight • Presence of a water pond (25 L plastic container, 60 × 39 × 16 cm)	• Ø • Ø • L 19 m	• Ø • Ø • Ø	Dispersal assessed after 5 days, with daily observations	• Biological model: White-legged damselfly *Platycnemis pennipes*	• Narrow S-shaped dispersal corridor, dark and warm • Resource limitation • Predatory cue (visual, auditive and olfactory = frogs)	[78]
• 130 L • Thin layer of soil and soil litter • Low (small piece of vegetable and 2 pieces of grass) or high food resources (half of a potato, half of a carrot, half of an apple and a handle of grass)	• 130 L • Thin layer of soil	• Plastic • ID 100 mm • L 4.4 m	• 16 to 25 °C • Ø • Ø	Dispersal assessed after 5 days, with daily observations every day	• Biological model: marsh cricket *Pteronemobius heydenii*	• Thin layer of soil in the container • Resource limitation • Predatory cue (olfactory cue = lizards)	[78]
• 50 mm; 65 mm; • no food material	• 50 mm; 65 mm; • 20 g of unbleached organic flour or rice	• Plastic • ID 5 mm • L from 250 to 1750 mm	• 30 °C • 65% • 14:10 (L:D)	Dispersal assessed after 48 h	• Biological models: Red flour beetle *Tribolium castaneum* and lesser grain borer *Rhyzopertha dominica*	• Distance among the containers (250, 750 and 1750 mm)	[79]
• 60 mm; 40 mm • wheat bran and one piece of carrot	• 60 mm; 40 mm • 0.5 cm of sand at the bottom of the container	• Plastic • ID 13 mm • L 1.6 m or 2.4 m	• 18 or 25 °C • 50% • 14:10 (L:D)	Dispersal assessed after 8 h and 24 h	• Biological model: Lesser mealworm *Alphitobius diaperinus*	• Angle of 15° for the tube connecting the containers • Resource limitation	This study

**Table 2:** Overview of potential manipulations of the environment of interest in experimental studies of insects’ dispersal. Some of the conditions can be manipulated to test the effects of prenatal and postnatal habitat conditions. For the tested insects, the knowledge of their foraging area (routine movements) is particularly helpful, even if this information might be difficult to obtain. Tentative predictions of possible effects of the conditions on individuals’ phenotypes and dispersal are illustrated with published examples.

Variable of Interest	For the Insect, the Variable Has an Effect on	Expected Effects on Dispersal
**Manipulation of the social environment**		
Sex ratio in the initial container (patch)	Likelihood to find a mate, likelihood of sexual reproduction	Effects on dispersal propensity and emigration rate [85]
Number of insects in the initial container (patch), population density	Level of intraspecific competition	Effects on dispersal propensity and emigration rate (density-dependent dispersal) [86]; Increased dispersal distance [87]
Reproductive status, age of the insects	Motivation to find a mate, behavior of males and females, deterioration of the physiological condition with aging	Effects on dispersal distances [88], effects on emigration rate (but also depends on the availability of trophic resources) [89], effects on successful immigration [80]
Level of relatedness, consanguinity	Kin competition, inbreeding avoidance	Increased dispersal distance [4,87]
**Manipulation of the biotic environment**		
Presence of predatory cues (chemical, visual, olfactive)	Behavior, personality	Effects on dispersal propensity and emigration rate (but may depend on the population density and body condition) [90,91,92]; Increased dispersal distance [93]
Quality of the trophic resources in the initial container (patch)	Fecundity, longevity, resistance to environmental stress	Effects on dispersal propensity and emigration rate [94] Effects on dispersal (flight) performance [95]
**Manipulation of the abiotic environment**		
Rearing temperature of the insects (Natal habitat effect)	Development, growth, and body size of the adult (smaller size of the dispersal appendices, lower amount of body reserves)	Effects on mobility; Lower temperatures may increase dispersal propensity (temperature gives information of the thermal environment that would be encountered by the adult) [96]
Resource quality when rearing the strain (Natal habitat effect)	Development growth, body size and physiological condition of the adult	Decreased dispersal distances and decreased immigration success as insects are more susceptible to dispersal mortality [97]; Decreased emigration rate [98]; Condition-dependent dispersal [60]
Temperature of the dispersal assay	Aerobic metabolism (energy production)	Depending on the temperature, increased or decreased dispersal speed [99]
**Manipulation of the dispersal system**		
Size of the containers	Increased likelihood of tactile stimulation when using containers of small size	Increases dispersal propensity [100]
Nature of the hostile matrix (shaded, dark, slippery, colder/warmer than the patch, S-shaped, angle) (can also be referred to as “matrix permeability”)	Increases dispersal cost and difficulty → selects insect with specific behavioral, morphological, and physiological features allowing to overcome the hostility of the matrix	Increases dispersal difficulty during the transience phase [72,77,101]; Effects on dispersal capacity and success [77]
Length of the dispersal corridor (tubes connecting the containers), simulates fragmentation of available patches	Increases dispersal cost → selection of insects having the physiological features allowing to cover the inter-patch distance; Over time, progressive increased reluctance of individuals to disperse	Effects on dispersal capacity and success (emigration and mortality during transience should be higher when the length of the dispersal path is increased) [90,102]
Duration of the dispersal assay	Less mobile and foraging insects which may reach the destination container	Effects on amount of individuals that emigrate [72,77]
Habitat quality (Presence of oviposition sites in the initial container (patch), nature of the medium, etc.)	Poor reproductive values	Effects on emigration rate [103]

## Appendix A

In order to get a first overview of the variables of potential interest when designing an experimental system assessing dispersal propensity and performance in entomological studies, a short experiment was conducted for the purpose of this review. The biological model for this trial was the tenebrionid beetle *Alphitobius diaperinus*. Two containers (patches) of 110 mL were used, connected by a 1.6 m long plastic tube of 13 mm I.D. making an angle of 15° from the source container (containing wheat bran and one piece of carrot—used as an oviposition site for the species) to the destination container (containing a layer of 0.5 cm of sand at the bottom of the container). Before the containers were connected, the beetles were acclimated for 24 h in the source container at 18 °C. Then, the assay was run for 24 h at 18 °C, and 19–20 unsexed insects were placed in the source container. In this experiment, the numbers of dispersing, in transit, and resident individuals were 32 ± 5%, 24 ± 5%, and 43 ± 6% (the proportions were marginally significantly, ⲭ² = 5.37; 2 ddl; N=213; *p* = 0.07). In another test, the angle in between the two containers was removed, the effects of temperature (18 versus 25 °C), duration of the assay (24 versus 8 h), nature of the medium (sand versus wheat bran) and number of insects (20 versus 40 adults) in the source container were assessed. As expected, temperature had a prominent effect on the dispersal propensity, with almost 100% of the 20 beetles that dispersed at 25 °C, and many of them (about 16 insects over the 20) reached the destination container in all replicates (N = 7) after 24 h; by reducing the duration of the assay to 8 h, the number of beetles that reached the destination container was around 10 in all replicates. When assessing the effects of the number of beetles in the source container, we found that the proportion of dispersing insects was two times higher in assays that contained 40 beetles as compared with those having 20 beetles (note that in this assay, the distance in between the container was 2.4 m). Importantly, the nature of the medium used in the source container made no differences in dispersal rates.
